# Spurious Serum Hormone Immunoassay Results: Causes, Recognition, Management

**DOI:** 10.17925/EE.2022.18.2.141

**Published:** 2022-08-15

**Authors:** Glenn D Braunstein

**Affiliations:** Department of Medicine, Cedars-Sinai Medical Center, Los Angeles, CA, USA

**Keywords:** Anti-streptavidin antibody, biotin, heterophile antibodies, high-dose hook effect, hormone binding proteins, hormone isoforms, human anti-animal antibodies, immunoassay

## Abstract

For over 50 years, immunoassays have been extensively used to quantitate hormones in blood, other fluids and tissues. Each assay has its own sensitivity, specificity and other analytical components. Despite the differences between commercial products, these assays provide important clinical information about hormone levels in patients. However, inaccurate results can occur because of technical issues, as well as patient-specific factors that can interfere with immunoassay hormone measurements. The latter include excessive normal blood or serum components, the presence of cross-reacting substances, extremely high levels of hormones leading to the high-dose hook effect, and interference from a variety of endogenous factors such as human antibodies that interact with the assay components or high levels of biotin in the serum from exogenous ingestion. This article briefly reviews the sources and recognition of endogenous interference, and describes methods to determine the correct serum hormone concentration.

Clinicians depend on accurate, precise and reproducible hormone measurements for the diagnosis and management of endocrine disorders. Immunoassays have constituted one of the major methods of measuring hormone levels in blood, urine and other fluids, as well as in tissue for over 50 years. The two major methodologies are competitive and non-competitive immunoassays, and each has multiple variations. Many technical (exogenous) issues can lead to inaccurate results, such as failure to collect samples in the correct tube or transport them at the appropriate temperature, but these are often detected through laboratory quality control measures.^1^ However, there exist a variety of endogenous factors specific to the individual or their sample that may result in an apparent, but spurious, abnormal hormone concentration (*Table 1*). Awareness of these potential causes of a result that does not fit the clinical findings or prior hormone measurements in the patient is important in order to avoid unnecessary additional tests or treatment for a situation that is misdiagnosed because of an inaccurate hormone measurement.

As illustrated in *Figure 1A*, in a competitive immunoassay, an anti-hormone antibody is bound to a solid surface such as a tube or bead. The patient’s serum containing the endogenous hormone and a small (‘tracer’) amount of the hormone of interest labelled with radioactive isotope, a dye, enzyme, metal or other substance that can be easily quantitated is mixed with the antibody and the serum. After incubation, the non-antibody-bound endogenous hormone and labelled hormone are washed away and the amount of labelled hormone bound to the capture antibody is measured. In some competitive immunoassays, the anti-hormone antibody is not immobilized on a solid surface; rather it is mixed directly with the endogenous hormone and labelled hormone, followed by precipitation of the anti-hormone antibody–hormone complex with a second antibody directed against the anti-hormone antibody (e.g. the first antibody may be derived from a rabbit and the second anti-rabbit immunoglobulin antibody made in sheep). Next, a wash step removes unbound endogenous and labelled hormone and then the precipitated labelled hormone is measured. As the labelled hormone and the endogenous hormone compete for the limited amount of antibody, the greater the concentration of the endogenous hormone, the less labelled hormone will be bound. Conversely, if there is a low concentration of endogenous hormone in the serum, there will be more binding of the labelled hormone. This results in an inverse dose–response curve, established with known quantities of purified hormone, with lower measured levels of the labelled hormone indicating a higher concentration of the endogenous hormone (*Figure 1B*).

In a non-competitive (‘sandwich’) immunoassay, two separate antibodies against the hormone are used, each directed to a different antigenic site on the hormone (*Figure 2A*). One antibody is bound to a solid surface to capture the endogenous hormone in the patient’s serum. The other antibody has a label that can be read visually or quantitated with an instrument. Unlike the situation with a competitive immunoassay, both antibodies are present in excess amounts. The endogenous hormone present in the serum is captured by the bound antibody and the labelled antibody also binds to the hormone. The higher the endogenous hormone concentration, the greater the signal from the labelled antibody, resulting in a direct, linear dose–response curve (*Figure 2B*).

**Table 1:** Endogenous factors interfering with hormone immunoassay measurements

1. Excessive normal blood or serum components			
a. Hyperlipidaemia	b. Hyperbilirubinaemia	c. Haemolysis	d. Hormone-binding protein alterations or abnormalities
2. Cross-reacting substances			
a. Exogenous hormone or medication	b. Hormone isoforms and subunits	c. Complex hormone–protein interactions	
3. High-dose hook effect			
4. Interference with assay components			
a. Autoantibodies	b. Heterophile antibodies	c. Human anti-animal antibodies	d. Anti-ruthenium antibodies
e. Anti-streptavidin antibody	f. Biotin		

**Figure 1:**
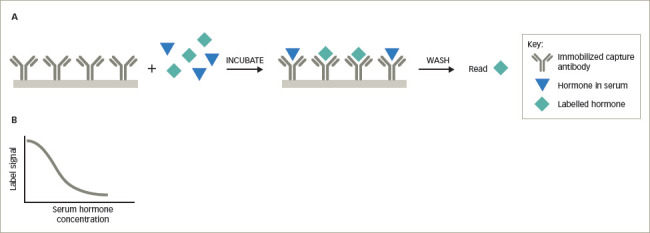
Competitive immunoassay

*A: Unlabelled hormone in the serum is mixed with a tracer amount of labelled hormone, both of which compete for binding to an immobilized antibody against the hormone. After washing away any unbound hormone, the label is quantitated (‘read’). B: The hormone concentration in the serum is calculated from a standard curve generated against known concentrations of the purified hormone.*

**Figure 2:**
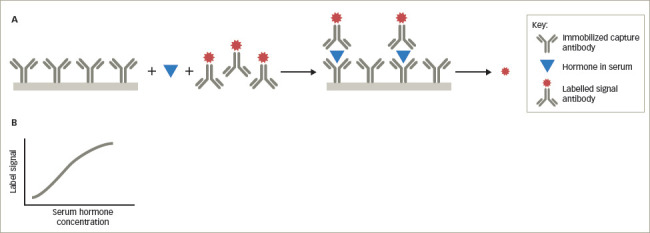
Non-competitive (‘sandwich’) immunoassay

*A: Serum is mixed with two antibodies directed against different epitopes on the hormone molecule. One antibody serves to capture the hormone and the other is labelled. Both bind to the hormone in the serum, forming a ‘sandwich’. The label present on the antibody that binds to the hormone–immobilized antibody complex is quantitated. B: The degree of labelled antibody binding to the hormone and immobilized antibody is directly proportional to the hormone concentration in the serum.*

## Excessive normal blood or serum components and transport protein abnormalities

One source of interference in some immunoassays is abnormal concentrations of normal blood constituents including lipids, bilirubin, nonesterified free fatty acids, haemoglobin and proteolytic enzymes released from red blood cells during haemolysis.^2,3^ These effects are often specific to certain assay components or method of measurement and may lead to falsely high or low concentrations, as many assays are dependent on using a plate reader and colour or turbidity could interfere with the measurement. A fresh blood specimen should be used in place of a haemolysed specimen. Turbid serum from hypertriglyceridaemia should either be ultracentrifuged or treated with lipase, or a fresh sample obtained after a 12-hour fast. If a specific assay is known to be affected by hyperbilirubinaemia, then either another immunoassay that is not altered by elevated bilirubin concentrations should be chosen, or, for some analytes such as steroid hormones, another method such as tandem mass spectroscopy could be used.^2^

Thyroxine-binding globulin binds most of the circulating thyroxine (T4) and triiodothyronine (T3); sex hormone-binding globulin does the same for the main sex steroid hormones, and cortisol-binding globulin for glucocorticoids. Congenital or acquired deficiency or excess of the binding proteins, or binding abnormalities due to gene mutations or medication interference can result in low or high concentrations of the total (proteinbound and -unbound) hormone. In these instances, measurement of the free or unbound hormone concentrations or calculation of the approximate free hormone concentration from measurement of the total hormone level and the binding protein concentration(s) will provide the correct information concerning the circulating hormone concentration.^4,5^ An exception is the artefactual increase in free T4 after the administration of heparin, which increases the concentration of non-esterified fatty acids that can displace free T4 (and free T3) from thyroxine-binding globulin. Immediate measurement of free T4 in a blood sample obtained 10 or more hours after the patient last received heparin will reduce this artefact.^4^ Individuals with mutations in the gene encoding for albumin may exhibit familial dysalbuminaemic hyperthyroxinaemia (FDH) and hypertriiodothyroninaemia with elevated concentrations of total T4 and T3, but usually have normal concentrations of free hormone when measured by equilibrium dialysis or ultrafiltration, as well as normal thyroid-stimulating hormone (TSH) levels. However, immunoassays that measure free T4 and free T3 using analogue methods may also exhibit spurious elevations in FDH.^6^

## Cross-reacting substances

The specificity of the antibodies used in an immunoassay dictates the ability of the assay to detect only the analyte of interest. In contrast to older assays that used polyclonal antibodies, the monoclonal antibodies used in today’s assays are more specific. Nevertheless, there may be crossreaction in some assays with endogenous or exogenous hormones, as well as medications. For instance, 17-hydroxyprogesterone, 21-deoxycortisol, 11-deoxycortisol, fludrocortisone and prednisolone cross-react in some commercial cortisol immunoassays.^7–9^ Anabolic steroids and some androgenic hormones such as dehydroepiandrosterone sulphate may cross-react in some testosterone immunoassays.^5,8^

Immunoassays may differ in their ability to detect various hormone isoforms. These differences account for the discrepancies between growth hormone concentrations measured in a 22K-growth hormonespecific assay versus an assay that measures both the 20K and other non-22K isoforms.^10^ The clinical use of pegvisomant, a growth hormone analogue that acts as a competitive receptor antagonist and is approved for the treatment of acromegaly, can also result in inaccurate results, and depending on the assay, the result may be high or low.^11^

Assay specificity issues have been well described for human chorionic gonadotropin (hCG). hCG is composed of two subunits, alpha and beta. The alpha subunit is homologous to the alpha subunits of the other glycoprotein hormones, follicle-stimulating hormone, luteinizing hormone and TSH. The beta subunit of the glycoprotein hormones determines the biological activity of the intact (alpha + beta) hormone. After the first few weeks of pregnancy, intact, biologically active hCG is the dominant moiety in the serum. Non-trophoblastic tumours may produce the free beta subunit of hCG. Assays that can detect all forms of hCG are useful for pregnancy diagnosis and for tumour screening or monitoring, while those that are highly specific for intact hCG are sufficient for pregnancy, but not for tumour detection or monitoring.^12^ Therefore, it is important to know whether one is ordering an intact hCG assay or a total-beta hCG assay in different clinical settings.

## High-dose hook effect

**Figure 3:**
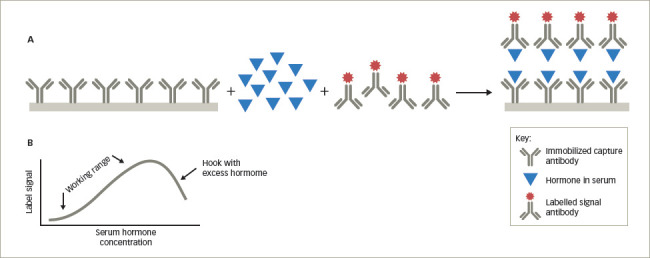
High-dose hook effect

As noted above, non-competitive or sandwich immunoassays contain two antibodies directed against different epitopes of the hormone, and in the presence of the hormone, both bind to the hormone forming a sandwich. In some assays, extremely elevated levels of the hormone may saturate both the first and second antibodies and prevent the formation of a sandwich. This results in a falsely low level of the hormone (*Figure 3A*). In the dose–response curves for assays where this is a problem, one will see the linear direct correlation between the concentration of the hormone and the signal generated from the labelled antibody in the working range of the hormone concentrations in the serum. However, when the hormone concentration exceeds this range, the dose–response curve ‘hooks’ down giving the falsely low results (*Figure 3B*). The major endocrine assays in which this is an issue are prolactin in patients with macroadenomas, hCG in patients with hydatidiform moles or choriocarcinoma, calcitonin with metastatic medullary carcinoma of the thyroid, and thyroglobulin in patients with metastatic differentiated thyroid carcinoma.^7,8^ When this phenomenon is suspected, the clinician should request that the laboratory repeat the hormone measurements on serial dilutions of the serum sample. In samples with markedly elevated hormone levels, the concentration of the hormone in the diluted samples will be higher than in the original serum sample.

## Interference with assay components

### Autoantibodies

Patients with autoimmune disease such as systemic lupus erythematosus may develop autoantibodies to hormones, as can individuals without known autoimmune conditions. Many case reports and case series have examined this problem for prolactin and TSH. Macroprolactin is a complex composed of prolactin and an autoantibody directed against the hormone.^7,8^ It is biologically inactive but is detected in prolactin immunoassays and may present a false elevation of prolactin. Different assays react differently to macroprolactin. When suspected, the laboratory can measure total serum prolactin and, following precipitation of the macroprolactin with polyethylene glycol, repeat the measurement to determine the monomeric prolactin concentration in the sample. Alternatively, gel filtration chromatography will separate the macroprolactin from monomeric prolactin. Less common is the finding of macrothyrotropinaemia (macro-TSH) due to an autoantibody to TSH complexed to TSH.^4,7,8^ As with macroprolactin, macro-TSH has low bioactivity, but can give falsely elevated TSH levels in TSH immunoassays. A clue to the presence of macro-TSH is the marked elevation of TSH in the presence of normal concentrations of T4 and free T4.^4^ Gel filtration chromatography is superior to polyethylene glycol precipitation for diagnosing the presence of macro-TSH.^7^ Other polypeptide hormones affected by autoantibodies include insulin, hCG, luteinizing hormone and follicle-stimulating hormone.^8,13^

Anti-thyroglobulin antibodies in patients with autoimmune thyroid disease interfere in thyroglobulin immunoassays and, depending upon the assay, may result in falsely low or elevated levels.^8^ Autoantibodies against T4 and T3 have also been reported, although they are uncommon.^4^ In one-step competitive immunoassays in which all the reagents are mixed, the autoantibodies bind to the labelled thyroid hormone analogue and less labelled analogue binds to the capture antibody. After the washing step, the labelled analogue bound to the autoantibody is washed away, resulting in a falsely low reading of the labelled analogue in the tube, leading to an apparent elevated concentration of the thyroid hormone. This is not an issue in a two-step assay in which the patient’s serum is mixed with the binding antibody, followed by a wash step and then the addition of the labelled T4 or T3 analogue tracer, as the autoantibody is washed away in that setting.

*A: In a non-competitive assay, extremely high concentrations of the hormone in the serum sample may result in binding of the hormone independently to each of the antibodies, preventing the antibodies from forming a ‘sandwich’. B: This results in a low reading of the labelled antibody and, thus, a spuriously low hormone concentration quantitated from the dose–response curve.*

**Figure 4:**
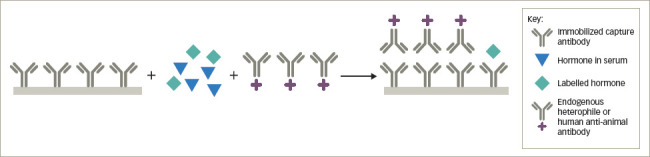
Heterophile or human anti-animal antibody interference in competitive immunoassays

*The heterophile or anti-animal antibody binds to the immobilized antibody, preventing the labelled hormone from binding to the immobilized capture antibody, leading to a false elevation of the hormone concentration in the serum.*

Another autoantibody, rheumatoid factor, found in patients with rheumatoid arthritis and other conditions binds to the Fc-domains in immunoglobulin G antibodies from a variety of species.^2,8^ In two-site immunoassays, rheumatoid factor may bind to both the capture and labelled antibody resulting in an apparent, but spurious, elevation of the hormone.

### Heterophile and human anti-animal antibodies

**Figure 5:**
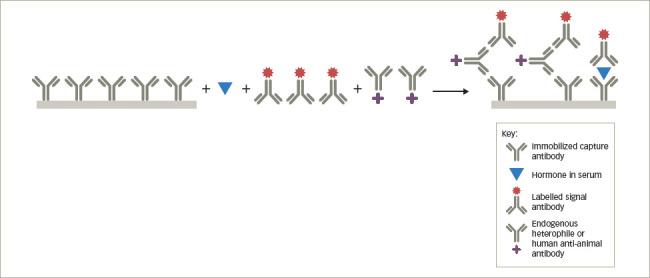
Heterophile or human anti-animal antibody interference in non-competitive immunoassay

Heterophile antibodies represent naturally occurring human antibodies that bind to anti-hormone antibodies produced in multiple different animal species, while human anti-animal antibodies refer to human antibodies formed against a specific animal species immunoglobulin from prior exposure to pets, workplaces that deal with animals or from exposure to animal monoclonal antibodies used therapeutically.^2,4,7,8^ The most common anti-animal antibodies are human anti-mouse antibodies. By binding to the capture antibody in a competitive immunoassay, they may sterically hinder the binding of the labelled hormone tracer giving a falsely high result (*Figure 4*). In a sandwich assay, the heterophile or anti-animal antibody may bind to both the capture and labelled antibody and, thus, mimic the presence of the hormone, also leading to a falsely elevated result (*Figure 5*). In some instances, the interfering antibody only binds to one of the antibodies and may lead to falsely low results. These antibodies have been shown to interfere with immunoassays for TSH, hCG, luteinizing hormone, follicle-stimulating hormone, prolactin, calcitonin, oestradiol, progesterone, testosterone, free T4, free T3, adrenocorticotrophic hormone, cortisol, parathyroid hormone and inhibin.^7,14–16^

### Anti-streptavidin and anti-ruthenium antibodies

**Figure 6:**
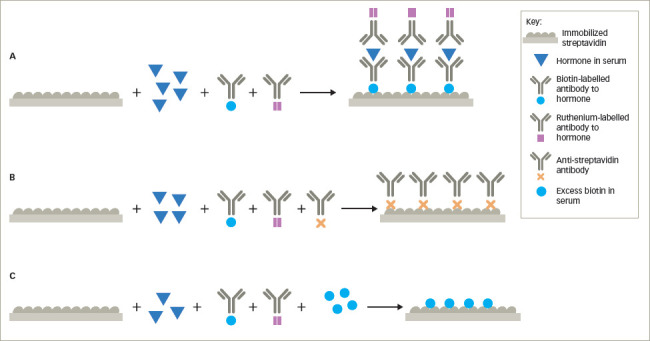
Effect of anti-streptavidin or anti-ruthenium antibodies or excess biotin in non-competitive immunoassays using binding of biotin to streptavidin as assay components

**Figure 7:**
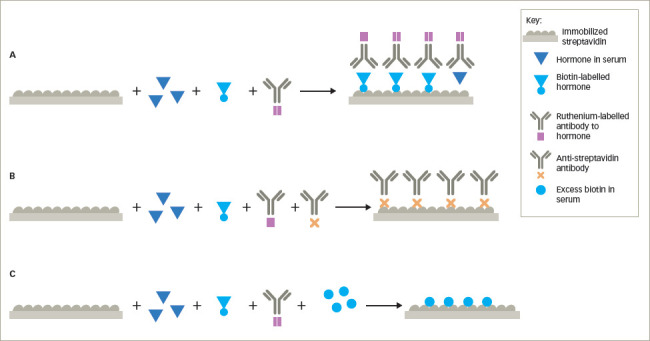
Effect of anti-streptavidin or anti-ruthenium antibodies or excess biotin in competitive immunoassays using binding of biotin to streptavidin as assay components

Biotin, also known as vitamin B7, is used extensively in immunoassays because it can be covalently attached to proteins, peptides, thyroid and steroid hormones without altering the antigenic activities of the hormones. Biotin strongly binds non-covalently with streptavidin, allowing biotin and streptavidin to be used in both competitive and non-competitive immunoassays. Some automated immunoassays utilize streptavidin bound to microparticles in a non-competitive format. Biotin-labelled monoclonal antibodies against the hormone of interest are mixed with the microparticles and the patient’s serum. The hormone in the serum binds to the biotin-labelled antibody and because of the affinity of biotin for streptavidin, the biotinylated antibody–hormone complex attaches to the streptavidin-coated particle. A second antibody against the hormone, often labelled with ruthenium is used as the signal antibody. After washing, tripropylamine, an electron emitter, is added and the electrons excite the ruthenium, which in turn emits photons measured as chemiluminescence, which is proportional to the amount of hormone bound to the particle (as a streptavidin–biotinylated antibody–hormone–ruthenium-labelled antihormone antibody complex) (*Figure 6A*). In a sandwich format, anti-streptavidin antibodies inhibit the binding of the biotinylated antibody–hormone complex from attaching to the streptavidin, leading to spuriously low hormone measurements (e.g. TSH) (*Figure 6B*). In competitive immunoassays, biotin-labelled hormone or hormone analogue compete for binding with a ruthenium-labelled antibody to the hormone, which binds to the streptavidin-coated particle through the biotinylated hormone (*Figure 7A*). Anti-streptavidin antibodies prevent this binding, resulting in false elevation of the hormone levels (e.g. free T3) (*Figure 7B*).^8,17–19^ The origin of anti-streptavidin antibodies is unknown, but may be due to prior exposure to streptavidin or the soil bacterium, *Streptomyces avidinii*, from which the protein is purified.^19^

*By binding to both the capture and labelled antibodies, the heterophile or anti-animal antibody results in an apparent high concentration of the hormone in the serum.*

Anti-ruthenium antibodies directed to either ruthenium or the ruthenium-labelled antibodies may result in an increase or a decrease in the chemiluminescent signal, which in turn may lead to inaccurately elevated or decreased hormone results depending on the interfering antibody specificity and assay format.^7,20^

### Biotin interference

The recommended daily intake of biotin is 30 μg, and at this intake, the serum levels range from 0.1 to 0.8 ng/mL, which is below the levels that can interfere in immunoassays. However, high-dose biotin is available as over-the-counter formulations for skin, hair and nail health, as well as several medical conditions. Large amounts of exogenous biotin may compete with the biotinylated antibodies, hormone or hormone analogue for the streptavidin binding sites (*Figure 6C*, *Figure 7C*). In competitive immunoassays, this generally results in spurious elevation of the hormone concentration, while in sandwich assays, this usually results in abnormally low levels of measured hormone.^21–23^

## Approach to identifying spurious hormone measurements

The first indication that a hormone measurement may be wrong is a discrepancy between the clinical picture and the hormone level, especially if prior measurements of the same hormone were stable but different from the current measurement. In that situation, it is best to repeat the measurement in a fresh sample after an overnight fast, which eliminates some of the pre-analytical factors such as a mislabelled specimen and reduces the potential for lipaemia. If the repeat study gives the same result as the first, then the laboratory staff should be asked to examine the serum specimen, which most laboratories will keep for 7 days after receipt, and to check whether the specimen was haemolysed, exhibited hyperbilirubinaemia or was lipaemic. If haemolysis was present, another carefully collected blood sample is required, with rapid separation of the serum from the cellular elements. Lipaemic samples can be ultracentrifuged or treated with lipase. Hyperbilirubinaemic serum can be evaluated in another assay, if elevated bilirubin is known to interfere with the initial method.

The patient should be questioned about biotin use. If positive, the biotin supplement should be discontinued for 2 days and the patient retested, as this will eliminate interference in assays that utilize streptavidin and biotin.^21,23^ Similarly, if there is a concern about an exogenous source of a cross-reacting substance, either the medication or supplement can be stopped for 5 half-lives of the cross-reacting moiety and a fresh serum specimen retested.

Suspected presence of macroprolactin and other autoantibody protein–hormone complexes can be confirmed through polyethylene glycol precipitation of the macromolecular complexes followed by remeasurement of the monomeric hormone in the residual serum, or separation via column chromatography.

*A: Reaction without interference. The biotin-labelled antibody to the hormone binds the hormone in the serum and both are bound via the biotin to the immobilized streptavidin. The second antibody, labelled with ruthenium or other type of label binds to the second site on the hormone. The label is quantitated, and the hormone concentration is directly proportional to the label signal. B: Anti-streptavidin antibodies interfere with the binding of the biotinylated antibody to the streptavidin, preventing binding of the hormone and the labelled antibody to the immobilized streptavidin. C: Excess biotin in the serum sample may saturate the streptavidin binding sites, preventing the biotinylated antibody hormone-labelled antibody from being immobilized, resulting in falsely low levels of measured hormone.*

*A: Reaction without interference. The biotinylated hormone competes with hormone in the serum for binding to the ruthenium-labelled antibody. Only the biotinylated hormone– ruthenium-labelled antibody complex binds to the immobilized streptavidin. The greater the amount of hormone in the serum, the less the amount of biotinylated hormone– ruthenium-labelled antibody complex that is bound to the streptavidin. B: Anti-streptavidin antibodies interfere with the binding of the biotinylated hormone–ruthenium-labelled antibody complex to streptavidin, resulting in spurious elevation of hormone levels in the serum. C: Excess biotin in the serum sample may saturate the streptavidin binding site, preventing the biotinylated hormone-labelled antibody from being immobilized, resulting in falsely high levels of measured hormone.*

The high-dose hook effect can be uncovered by having the laboratory perform serial dilutions of the serum. As the specimen becomes more dilute, the amount of the hormone in the sample will eventually fall within the working range of the assay and further dilutions will give comparable results (see *Figure 3B*).

Assay interference by heterophile, human anti-animal antibodies, anti-ruthenium and anti-streptavidin antibodies can be identified by several techniques including re-measuring a sample in another assay platform that utilizes different reagents or methodology (e.g. liquid chromatography–tandem mass spectroscopy), adding heterophile- or immunoglobulin-blocking reagents to the assay, or performing serial dilutions of the sera, as the apparent concentration will often increase as the serum becomes more dilute.^24,25^ In addition, for some hormones such as hCG, interfering antibodies can be inferred from finding elevated levels in the serum without hCG being found in the urine, as the interfering antibodies are too large to be excreted in the urine.^25^

A recent publication provides a useful algorithm that outlines an in-depth approach to the immunoassay interference investigations discussed above.^26^

## Conclusions

In summary, when the clinical picture does not correlate with the hormone measurement, speaking to the patient about use of interfering substances and consultation with the laboratorian about the approach to evaluating the problem are the essential steps in identifying the cause of the discrepancy and determining the true hormone value. Manufacturers of commercial immunoassays usually supply clinical laboratories with a table listing cross-reacting or interfering substances for specific assays. For the most common, or quantitatively most important, interfering substances, laboratory directors should be encouraged to include this information in the results reports that are returned to the ordering clinician.
